# The effect of lateral π-extension on azulene-based molecules on surface studied by LT-STM

**DOI:** 10.1038/s41598-026-46150-4

**Published:** 2026-04-02

**Authors:** Suchetana Sarkar, Natasha Khera, Kwan Ho Au-Yeung, Renxiang Liu, Ji Ma, Xinliang Feng, Francesca Moresco

**Affiliations:** 1 Center for Advancing Electronics Dresden, TU Dresden, 01062 Dresden, Germany; 2 Chair of Molecular Functional Materials and Faculty of Chemistry & Food Chemistry, TU Dresden, 01062 Dresden, Germany; 3 Max Planck Institute of Microstructure Physics, Weinberg 2, Halle, Germany; 4 Present Address: Department of Chemistry, Philipps-Universität Marburg, 35032 Marburg, Germany; 5 Present Address: Physikalisches Institut, Karlsruhe Institute of Technology, Karlsruhe, Germany

**Keywords:** Chemistry, Materials science

## Abstract

Azulene-based systems offer a robust platform for exploring molecular self-assembly and electronic properties on surfaces due to their non-benzenoid topology and dipolar structure. Here, we report the low-temperature scanning tunnelling microscopy investigation of two structurally related azulene-based molecules, namely cyclopenta[cd]azulene trimers (**CPAT**) and its phenyl extended analogue cyclopenta[cd]azulene trimers (**CPAT-Ph**). The latter has lateral extensions in the form of three phenyl rings, designed to investigate the influence of functionalization on supramolecular behaviour. We demonstrate that while **CPAT** self-assembles in homochiral domains on Au(111), **CPAT-Ph** is weakly adsorbed and mobile on Au(111) and on Cu(110), exhibiting no ordered domains. Spatially resolved scanning tunnelling spectroscopy (STS) reveals that lateral π-extension primarily modifies electronic level alignment through altered molecule–substrate coupling, while leaving intermolecular electronic interactions weak. Upon thermal activation, both molecules undergo disordered intermolecular linkages on Au(111). However, on Cu(110) enhanced interaction between the substrate and the **CPAT-Ph** molecules enables intramolecular cyclodehydrogenation through multiple competing ring-closure pathways. These findings indicate lateral π-extension as a design parameter for tuning adsorption-dominated electronic structure and surface reactivity in non-benzenoid π-systems.

**Supplementary Information:**

The online version contains supplementary material available at 10.1038/s41598-026-46150-4.

## Introduction

Non-benzenoid hydrocarbons represent an intriguing frontier in molecular design, offering access to electronic and structural properties not attainable in conventional benzenoid systems^1–3^. Among these, azulene is of particular interest due to its fused 5–7 ring topology, resulting in a distinct electronic structure characterized by a permanent dipole moment and a low-lying excited state^4,5^. These features have motivated studies in azulene derivatives across diverse applications, ranging from organic optoelectronics^6^ and nonlinear optics^7^ to supramolecular materials^8,9^. In general, subtle changes in functionalization can have pronounced effects on intermolecular interactions, conformational preferences, and ultimately on the behaviour of molecules confined to two-dimensional environments^10–13^. In particular, the inherent asymmetry and polarizability^14^ of the azulene core makes it a promising building block for exploring molecular organization at surfaces. Understanding how such molecules behave on surfaces is critical for the rational design of functional nanoscale systems, where precise control over assembly and orientation is essential. In this context, the on-surface synthesis of non-benzenoid nanographenes^15^, a class of polycyclic aromatic hydrocarbons with well-defined topologies, has attracted significant interest^16,17^. Nanographenes, which can be regarded as finite graphene segments on the nanometre scale, exhibit exceptional physicochemical properties^18,19^, and can enable direct integration of azulene units into extended π-conjugated systems^20^.While azulene derivatives are known for their structural and electronic versatility on surface, few studies have explored how peripheral substitution influences their self-assembly and surface reactivity.

In this work, we compare two azulene derivatives namely cyclopenta[*cd*]azulene trimers [**CPAT**] and its phenyl extended analogue cyclopenta[*cd*]azulene trimers [**CPAT-Ph**], to investigate how lateral π-extension influences intermolecular interactions, surface mobility and reactivity in non-benzenoid π-systems. **CPAT** and **CPAT-Ph** share the same azulene trimer core; the phenyl substituents in **CPAT-Ph** increase conjugation and steric bulk while introducing additional torsional degrees of freedom. These factors are known to modify adsorption strength and electronic level alignment on noble-metal surfaces^11^.

This comparative approach, combined with low-temperature scanning tunneling microscopy (LT- STM) and spectroscopy (STS) investigations, provides direct insight into how molecular architecture influences adsorption geometry, electronic structure, and thermal transformation pathways on metal surfaces. LT-STM has the ability to resolve submolecular features and probe electronic properties under ultrahigh vacuum (UHV) conditions making it ideally suited for studying complex systems like azulene-based molecules and nanoarchitectures, which often exhibit non-covalent interactions that are easily disrupted under ambient conditions^4,21,22^. Moreover, LT-STM enables the direct observation of domain formation, structure and intermolecular registry, factors that are central to understanding the behaviour of complex aromatic molecules at surfaces.

The chemical synthesis of both molecules has been detailed in reference^23^. While structurally similar, the difference being three additional phenyl rings in **CPAT-Ph**, we first show that this lateral π-extension results in a different chirality expression and adsorption geometry on Au(111). We then correlate these findings with electronic structure using STS. Finally, by comparing thermal activation on Au(111) and Cu(110), we show how molecule–substrate coupling determines access to intramolecular cyclodehydrogenation pathways towards non-benzenoid nanographenes.

## Results and discussions

### Adsorption of CPAT and CPAT-Ph on Au(111)

**Fig. 1:**
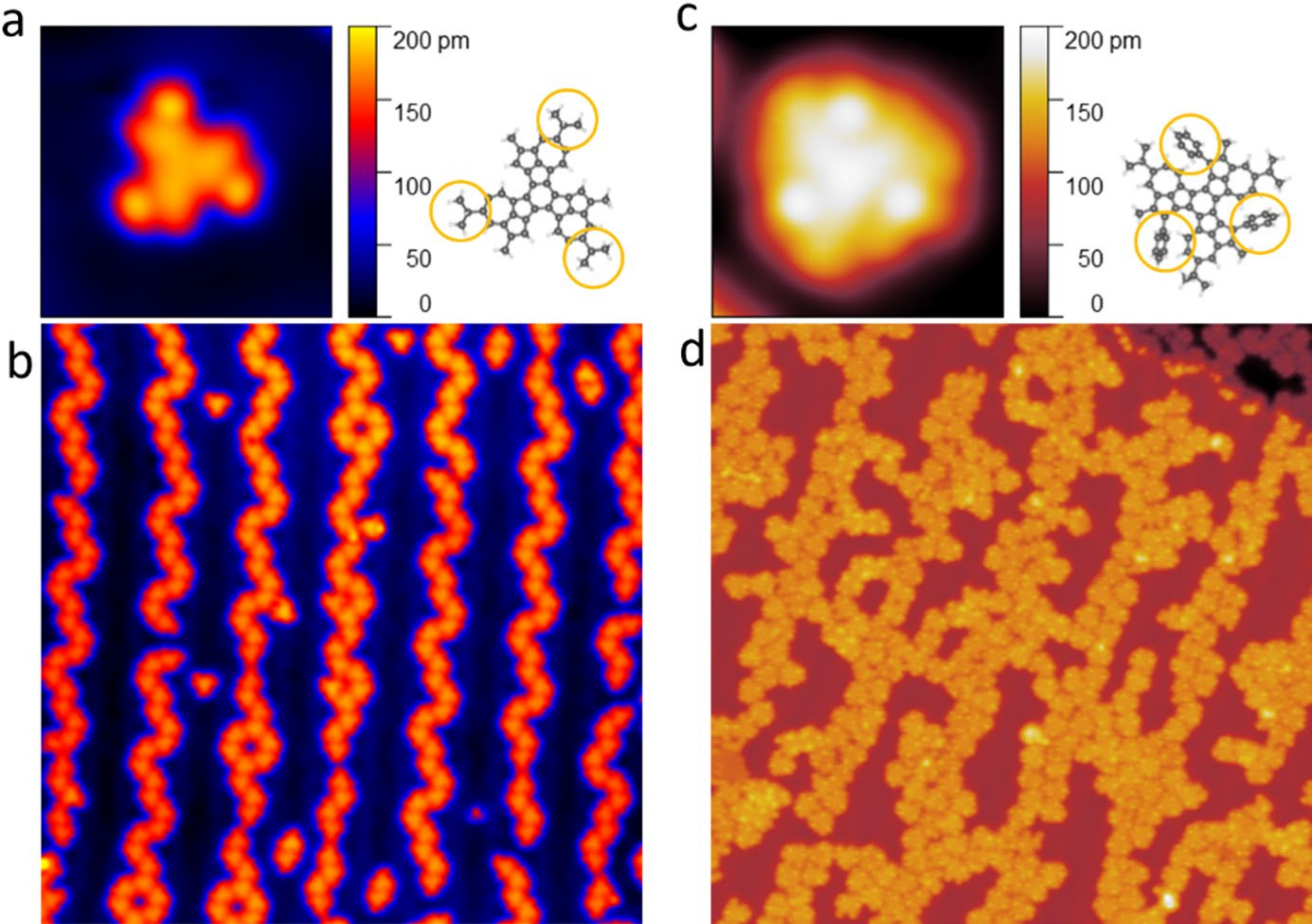
STM images comparing self-assembly and single molecule structure of **CPAT** and **CPAT—Ph** on Au(111). (**a**) Single molecule close-up of **CPAT** and its chemical structure. The yellow circles highlighting the isopropyl groups correspond to the lobes in the STM image. (**b**) Large area overview showing ordered self-assembled **CPAT** along the fcc direction. (**c**) Single molecule close-up of **CPAT—Ph** and its chemical structure. The yellow circles highlighting the phenyl groups correspond to the lobes in the STM image (**d**). Large area overview showing disordered **CPAT-Ph** with no preferential adsorption sites. Image parameters: (**a**), (**c**) V = 0.5 V and I = 20 pA; size 2.5 × 2.5 nm^2^ (**b**), (**d**) V = 0.5 V and I = 20 pA; size 40 × 40  nm^2^.

Both molecules were sublimated in ultra-high vacuum (UHV) conditions onto the Au(111) surface kept at room temperature. A comparison of their different adsorption behaviour has been shown in Fig. 1. The **CPAT** molecules (Fig. 1a,b) show a strong preferential adsorption on the fcc sites of the Au(111) reconstruction as typical for organic molecules on Au(111)^24,25^ (see also SI Fig. S1). An STM image of a single molecule is shown in Fig. 1a, along with the chemical structure. The yellow circles illustrate that the differentiated lobes can be attributed to the isopropyl side groups.

Contrastingly, the **CPAT-Ph** molecules (Fig. 1c,d) exhibit no self-assembly on the same surface, forming clusters across the herringbone reconstruction. The single molecule image (Fig. 1c) shows bright, differentiated lobes at the periphery, which can be attributed to the out of plane twisting of the phenyl groups, illustrated in Fig. 1c. Apart from the site-specific adsorption, the **CPAT-Ph** is also found to be more mobile on Au(111) as compared to the **CPAT**, possibly due to decreased molecule–substrate interaction.

**Fig. 2:**
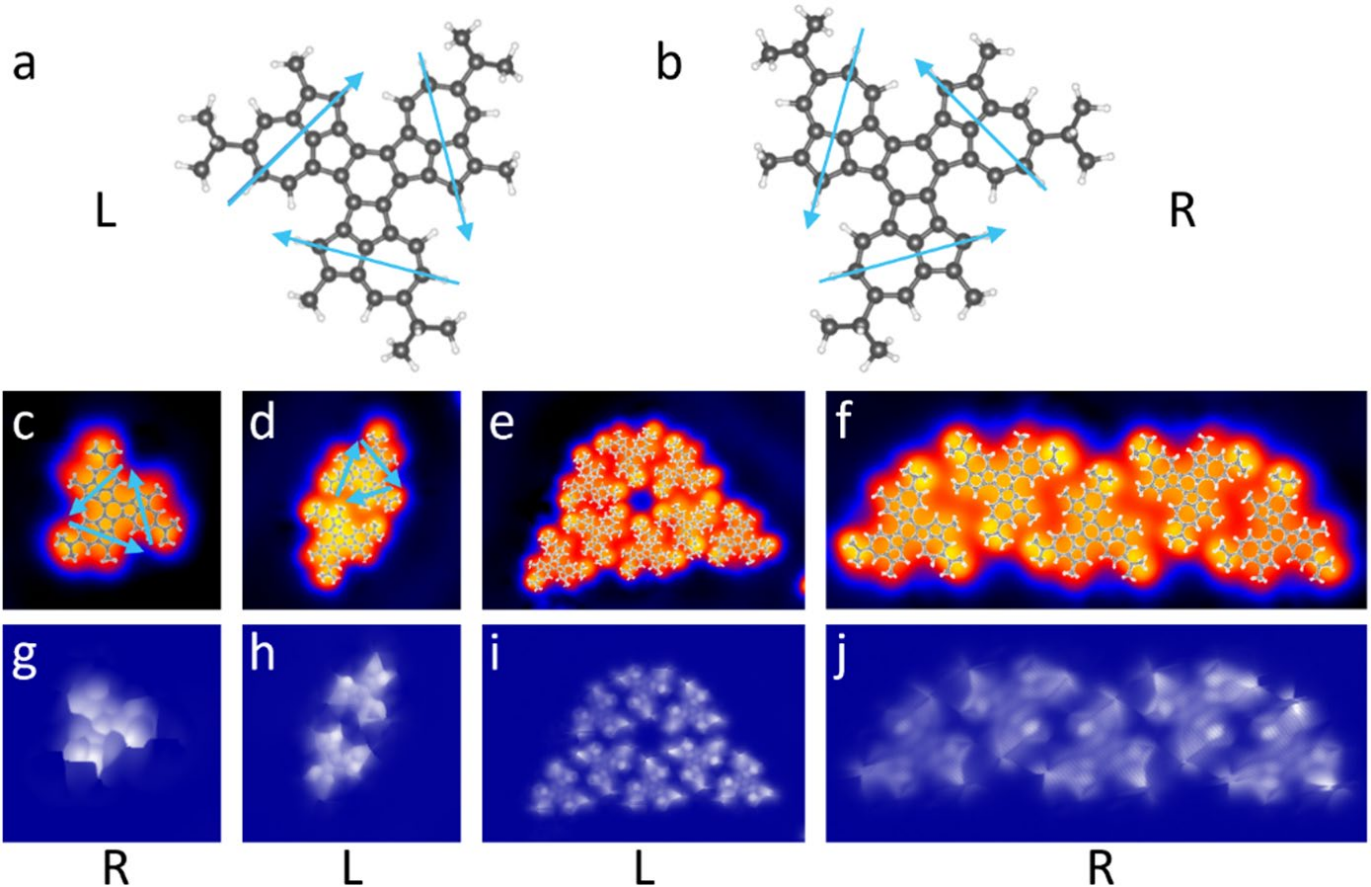
High-resolution STM images of self-assembled **CPAT** nanostructures and the two chiralities on Au(111). (**a**) and (**b**) The two different chiralities, L and R. The blue arrows show the orientation of the azulene units, which have been used to assign left-handed and right-handed chiral notations. (**c**)–(**f**) Constant current STM topographies of molecules arranged in a homo-chiral manner tentatively superimposed by molecular structures, as marked by L and R. (**g**)–(**j**) Corresponding constant height CO-tip images showing the molecular geometry. Image parameters: (**c**) V = 0.2 V; I = 20 pA; size 2.9 × 2.9 nm^2^. (**d**) V = 0.2 V; I = 20 pA; size 4.3 × 4.3 nm^2^. (**e**) V = 0.2 V; I = 20 pA; size 8.5 × 5.7 nm^2^. (**f**) V = 0.2 V; I = 20 pA; size 7.1 × 2.9 nm^2^. (**g**)–(**j**) V = -5 mV; size: (**g**) 2.9 × 2.9 nm^2^, (**h**) 4.3 × 4.3 nm^2^, (**i**) 8.5 × 5.7 nm^2^, (**j**) 7.1 × 2.9 nm^2^.

In Fig. 2 we consider the adsorption geometry of **CPAT** on Au(111), specifically the evidence of homochiral assembly. As schematically shown in Fig. 2a and b, the **CPAT** molecules can adsorb either in the left-handed chirality, marked by “L” in the figure or right-handed chirality, marked by “R” in the figure. We assign the L and R configurations based on the clockwise or counterclockwise orientation of the azulene units relative to the molecular centre, as indicated by the blue arrows in Fig. 2a and b. In the STM images of Fig. 2c–f we observe enantioseparation, *i.e*., the different self-assembled nanostructures are comprised of molecules with the same chirality, rather than a mix (see also SI Fig. S2). This separation of the two chiral conformers into homochiral domains is confirmed by bond-resolved constant-height CO functionalized tip images, as shown in Fig. 2g–j.

On achiral substrates such as Cu(111)^26^ or Au(111)^27,28^, intrinsically chiral molecules like **CPAT** can spontaneously separate into homochiral domains due to favourable non-covalent interactions, such as hydrogen bonding, van der Waals interactions, dipole–dipole interactions, and steric complementarity. It is also known that molecules with donor–acceptor characteristics or with dipoles also segregate into such homochiral domains on non-vicinal surfaces as well^29^. This enantioselectivity into one or the other conformation is an important building block of bio-materials, and is the subject of on-going study in molecular organics and therefore, single molecule insights are valuable^30^.

By comparing the adsorption geometry of the two molecules on Au(111), we can conclude that lateral π-extension weakens adsorption constraints on Au(111) as evidenced by the more mobile **CPAT-Ph**, suppressing both long-range order and spontaneous enantioseparation, as shown in Fig. 1 and in the SI (Fig. S3), This suggests that adsorption geometry and molecule–substrate coupling strength are the primary factor governing chirality expression in these non-benzenoid systems.

### Effect of lateral π-extension on electronic structure

**Fig. 3:**
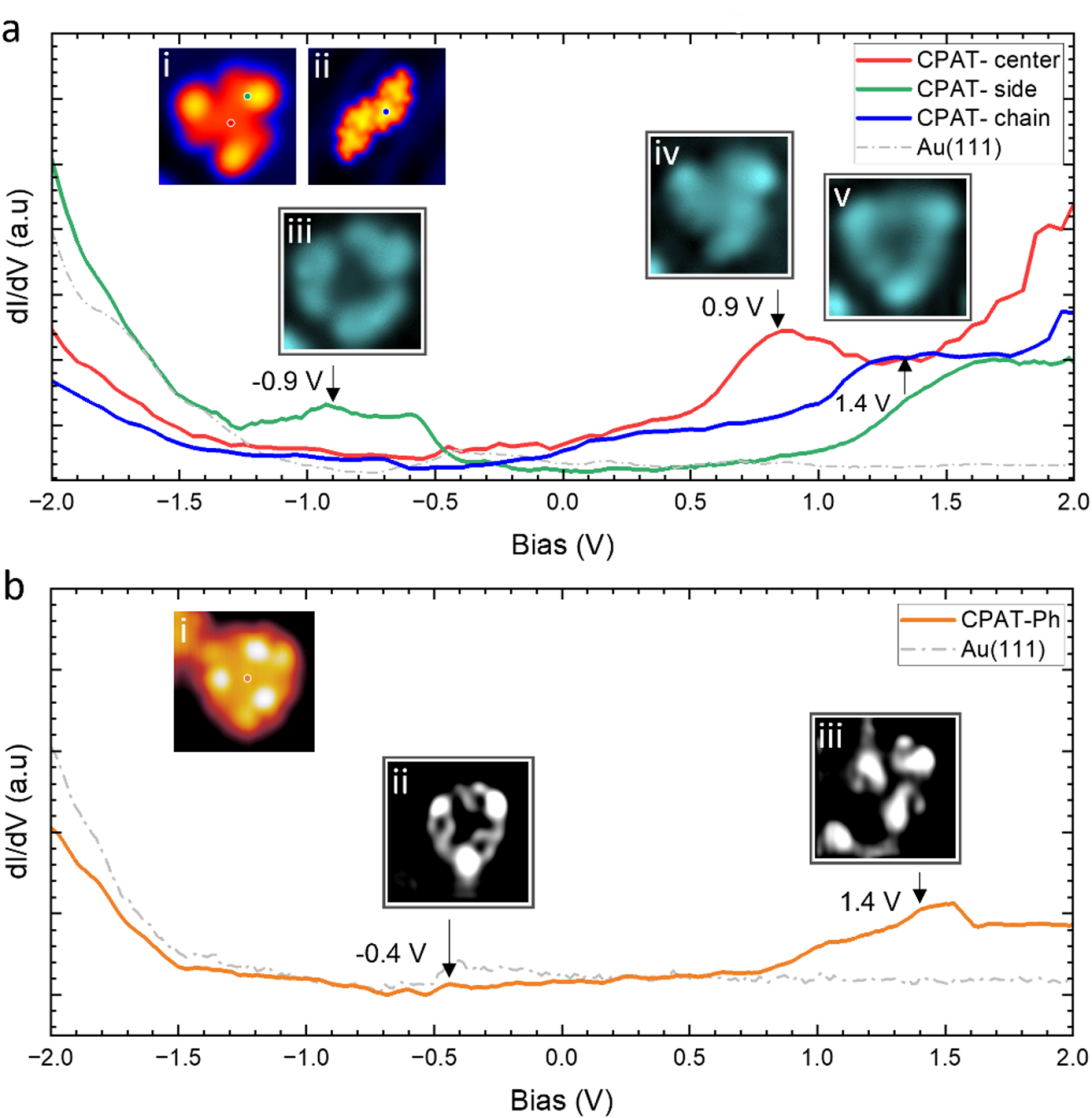
Comparison of electronic properties of **CPAT** and **CPAT-Ph** on Au(111). (**a**) dI/dV spectra on single **CPAT** molecule (red & green) and a **CPAT** in chain (blue). (i) STM image of single **CPAT** molecule. Image parameters: V = 0.2 V; I = 20 pA. (ii) STM image of **CPAT** molecule in chain. Image parameters: V = 0.2 V; I = 20 pA. (iii)-(v) Corresponding dI/dV maps taken at different biases obtained from STS. Image parameters: I = 200 pA; size (i), (iii), (iv), (v) 2 × 2 nm^2^. (ii) 5 × 5 nm^2^. (**b**) STS on a single **CPAT-Ph** molecule (orange). (i) Close up of a single **CPAT-Ph** molecule. Image parameters: V = -0.4 V; I = 30 pA. (ii), (iii) corresponding dI/dV maps taken at bias obtained from STS. Image parameters: I = 30 pA; size (i), (ii), (iii) 3.5 × 3.5 nm^2^.

In the second part of the experiment, we compared the STS measurements on both molecules (Fig. 3). For **CPAT** (Fig. 3a), differential conductance spectra recorded on isolated molecules (see also SI Fig. S4) and chains reveal a small peak at approximately V = -0.9 V and two peaks above the Fermi level, one at V = 0.9 V for the single molecule and V = 1.4 V for the molecule in a chain. The differential conductance maps taken at these peaks show the spatial distribution of the electronic resonances on a single molecule (Fig. 3a(i)).

For simplicity, we ascribe the resonance at V = −0.9 V to the Highest Occupied Molecular Orbital (HOMO), V = 0.9 V to the first Lowest Unoccupied Molecular Orbital (LUMO), and V = 1.4 V to the LUMO + 1 (see Methods for further details).

Although the dominant occupied resonance in our STS data appears more as a shoulder than as a sharp maximum, this is a common manifestation of molecular resonances on metal surfaces. Such broadened or asymmetric line shapes arise from hybridization with the continuum of substrate states and do not preclude assignment to molecular orbitals. Our assignment here is further supported by the spatial distribution of the local density of states distributions (LDOS) at this bias. The correspondence between resonances and LDOS via dI/dV maps allows unambiguous assignment of the observed features to the frontier molecular orbitals.

The spatially resolved dI/dV maps are shown in Fig. 3a(iii)–(v). The conductance map corresponding to the HOMO shows that the resonances are localised around the peripheral azulene units with the lowest intensity at the molecular core (Fig. 3a(iii)). The LUMO is delocalised over the entire molecule as shown by Fig. 3a(iv). The LUMO + 1 however, shows localization around the periphery and enhanced intensity at the isopropyl groups, as shown in Fig. 3a(v). Additional dI/dV maps acquired at these energies show that the corresponding LDOS are essentially identical for isolated and self-assembled homochiral nanostructures, be it dimers or chains (see SI Fig. S5), indicating a low interaction between the molecules.

In comparison, **CPAT-Ph** (Fig. 3b) exhibits a qualitatively similar electronic structure but with a notable shift of the occupied resonance toward the Fermi level. This shift reflects the influence of lateral π-extension on molecule—substrate coupling and electronic level alignment. Conductance maps taken of a single molecule (Fig. 3b(i) and SI Fig. S6) show that, despite the increased molecular size and conjugation length, the LDOS remains primarily localized on terminal azulene units, as shown in Fig. 3b(ii) and b(iii).

The comparison of STS spectra of the two molecules illustrates that the **CPAT-Ph** occupied states are shifted toward the Fermi level with respect to the **CPAT**. Furthermore, for **CPAT**, the electronic states remain nearly unchanged between monomeric and self-assembled forms, reflecting weak intermolecular electronic coupling. These results demonstrate that lateral π-extension modifies electronic level alignment while leaving intermolecular electronic interactions weak.

### Adsorption and thermal activation of CPAT-Ph on Cu(110)

Recent reports have shown that isopropyl-substituted precursors can undergo surface-assisted cycloaromatization to form polyarylene chains^31^ and nanographenes^32^. We therefore examined whether **CPAT** exhibits similar thermally induced cyclization or ring-closing reactions. Post-annealing the **CPAT** on Au(111) in steps up to 240 °C, we observed desorption and molecular linkages exhibiting no long-range order (see SI Fig. S7).

Annealing **CPAT-Ph** on Au(111), we observe that even at the elevated temperature of 300 °C, there is no significant desorption (see SI Fig. S8). This could be attributed to the phenyl ring extensions in **CPAT-Ph,** which causes a stronger interaction with the gold surface. As reported previously, oxidative ring-closing reactions leading to nanographene formation can occur at comparable temperatures^17^, supporting the plausibility of similar processes in our case.

**Fig. 4:**
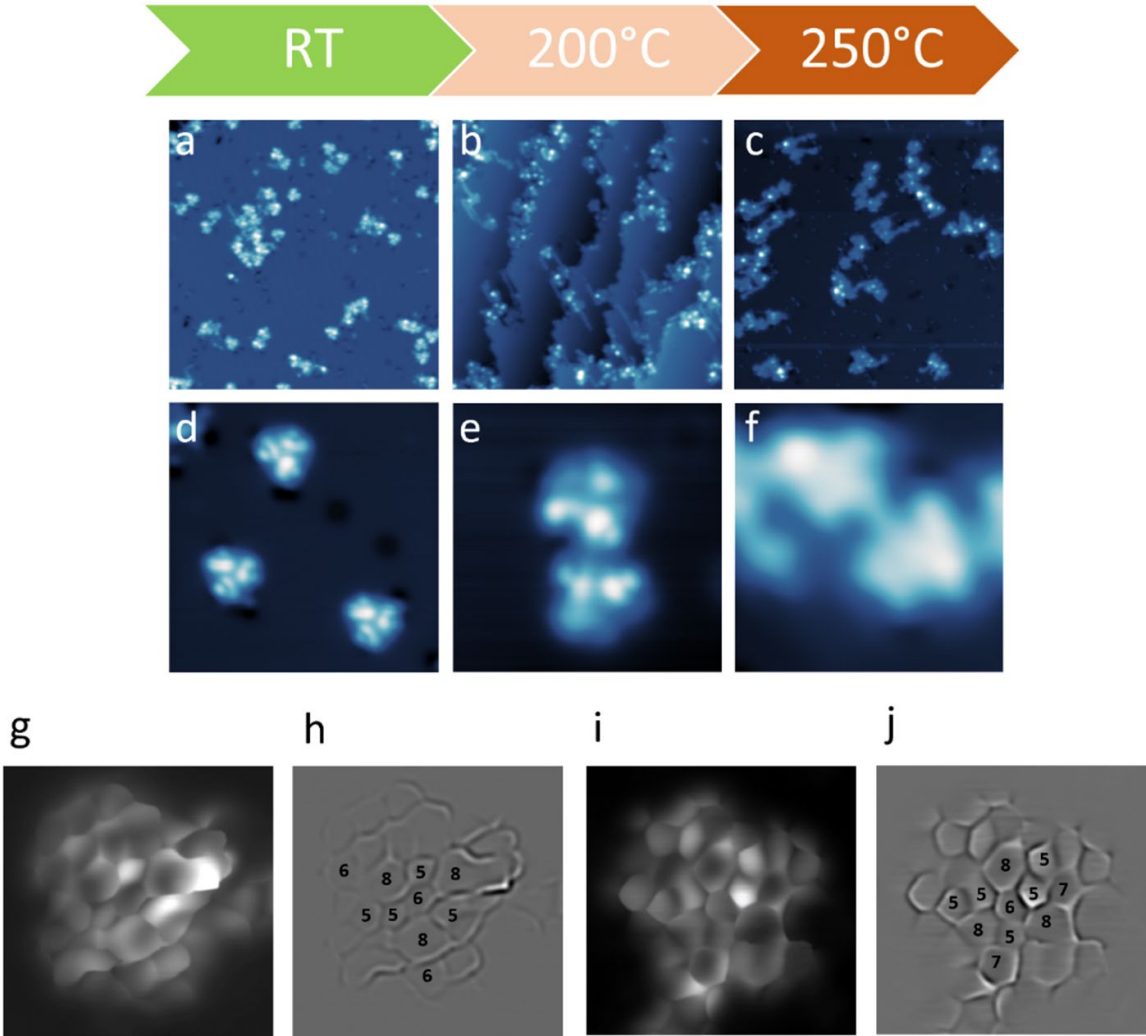
RT deposition and post-annealing **CPAT-Ph** on Cu(110). (**a**)–(**c**) Large area STM overviews after annealing the CPAT-Ph molecules on Cu(110) for 10 min at the specified surface temperatures. (**d**)–(**f**) Smaller area scans showing planarization and linkages. Image parameters: (**a**), (**b**) V = 0.2 V; I = 20pA, size 40 × 40 nm^2^, (**c**) V = 0.2 V; I = 10 pA; size 40 × 40 nm^2^. (**d**) V = 0.2 V; I = 20 pA; size 10 × 10  nm^2^. (**e**) V = 0.2 V; I = 50 pA; size 5.8 × 5.8  nm^2^. (**f**) V = 0.2 V; I = 50 pA; size 4 × 4  nm^2^. High-resolution images of two planarized **CPAT-Ph** molecules on Cu(110). (**g**, **h**) Constant height CO tip image and Laplace filter of molecule **1**, respectively. (**i**, **j**) Constant height CO tip image and Laplace filter of molecule **2**, respectively. Image parameters: (**g**, **h**, **i**, **j**) V = 6 mV; size 2 × 2 nm^2^.

Since we found the **CPAT-Ph** molecules to be more robust up to elevated temperatures, we deposited **CPAT-Ph** on the Cu(110) surface as it has been demonstrated that on the more reactive Cu(110) surface the intramolecular cyclodehydrogenation happens readily, suppressing polymer growth and favouring formation of discrete ring-closed molecules^33^. Additionally, Cu surfaces have the potential for stronger adsorption than Au surfaces, promoting stabilization of reaction intermediates^34,35^. After sublimation of **CPAT-Ph** on Cu(110) at low coverage, we observe a different adsorption geometry compared to the case of Au(111) (Fig. 4). The overview STM image (Fig. 4a) shows that there is no self-assembly also on Cu(110) and isolated molecules are visible on the surface. Figure 4d shows three isolated molecules, where the central ring appears with lower contrast, indicating that the molecules are not planar on this surface but adopt a more concave conformation compared to Au(111). This deviation from planarity can be attributed to a stronger interaction of the central ring of the molecule with the more reactive copper surface. To demonstrate this, we have also extracted line profiles on both substrates. This is shown in the SI (Fig. S8a,b).

Post-annealing of **CPAT-Ph** on Cu(110) induces molecular linkage and planarization at lower temperatures than on Au(111). The overviews in Fig. 4b and c show several oligomeric structures, both on step edges (Fig. 4b) and on larger terraces (Fig. 4c). In the latter, we can also observe the disappearance of some bright lobes within the aggregates. This is corroborated by smaller area scans presented in Fig. 4e and f, where along with linkages, it is also possible to discern the removal of some of the bright lobes, indicating a partial planarization.

Furthermore, we also find a few isolated molecules after annealing at 250°C, an example of which has been shown in the SI (Figs. S9 and S10). This contrasts with the results on Au(111), where we could mostly observe linear linkages. The single molecule also appears to be planarized, as signified by the disappearance of the bright side lobes, previously visible (Fig. 4d). Line profiles comparing single **CPAT-Ph** on Au(111), and pre- and post-annealed on Cu(110) are shown in the SI (Fig. S9c).

To understand the structure of these planarized molecular species, we performed bond-resolved high-resolution CO-tip constant height images. These are shown in Fig. 4g–j for two different molecules. Figure 4g shows closed rings of various orders, and its Laplace filtered image is shown in Fig. 4h. Here, we can start counting the ring order, and a few of them have been numbered for ease. The bright feature in Fig. 4g may be attributed to the 8-membered ring which is not fully cyclized or closed. The second molecule in Fig. 4i again shows planarization and ring closures, albeit with different ring orders. A few of these have been marked in the Laplace transformed image shown in Fig. 4j.

We conclude that we can obtain intermediate products to nanographenes, where single intramolecular ring-closing reactions happen, yielding fused ring systems of varying sizes. Given the multiple reactive C-H sites in **CPAT-Ph**, several intramolecular cyclodehydrogenation pathways are chemically plausible on Cu(110). These include phenyl-azulene fusion, azulene-azulene coupling, and phenyl–phenyl closure, yielding fused ring systems of varying sizes. The structural diversity observed in our high-resolution STM images is therefore correlated with the coexistence of multiple competing reaction pathways. An example of such ring closing reactions has been shown in the SI (Fig. S11). We emphasize that these schematics represent chemically plausible pathways consistent with the observed STM contrast and are not intended to imply a unique or exclusive reaction mechanism.

These findings suggest that while Cu(110) provides a more favourable surface environment for the formation of non-benzenoid nanographenes compared to Au(111), the resulting structures show different examples of ring-closing, giving rise to 5,6,7,8 membered rings. This structural variability is consistent with prior reports indicating that bottom-up on-surface synthesis of nanographenes frequently yields unanticipated and heterogeneous architectures^19^.

## Conclusions

By comparing **CPAT** and its laterally π-extended analogue **CPAT-Ph** on Au(111) and Cu(110), we show that lateral π-extension primarily tunes molecule–substrate coupling rather than inducing intermolecular electronic hybridization. Spatially resolved STS demonstrates that the frontier electronic states retain their molecular character irrespective of self-assembly, while enhanced coupling on Cu(110) enables thermally activated intramolecular cyclodehydrogenation through multiple competing pathways. These results suggest lateral π-extension as a practical handle for controlling adsorption-dominated electronic structure and surface reactivity in non-benzenoid π-systems.

## Methods

**CPAT** molecules were evaporated at 350 °C onto Au(111) surface kept at room temperature (25 °C), while the **CPAT-Ph** was evaporated at 320 °C. The **CPAT-Ph** molecules were evaporated onto a Cu(110) sample held at room-temperature. The coverage was monitored by a quartz microbalance to obtain for both molecules a coverage of about 0.5 monolayer. Before evaporation, the samples were cleaned by subsequent cycles of Ar^+^ sputtering and annealing to 450 °C. STM experiments were performed using a custom-built instrument operating at a low temperature of *T* = 5 K under ultrahigh vacuum (*p* ≈ 1 × 10^–10^ mbar). All shown STM images were recorded in constant-current mode with the bias voltage applied to the sample.

The differential conductance (dI/dV) spectra obtained in our LT-STM measurements provide a direct measure of the LDOS of the molecule–substrate system. In the tunnelling regime, the measured current $I\left(V\right)$ arises from the electron transmission between the tip and the sample^36^.

Spatially resolved dI/dV maps were acquired at bias voltages corresponding to the observed resonances. These maps reflect the spatial distribution of LDOS at the selected energy and hence visualize the real-space structure of the molecular orbitals involved in tunneling^37,38^. This combined spectral-spatial correlation provides strong evidence for assigning the resonances in the STS curves to the frontier molecular orbitals.

For spectroscopy experiments, all measurements were conducted in constant height mode. Spectra were measured using lock-in detection with a modulation frequency of 833 Hz and a modulation amplitude of 40 mV. Only metallic tips, i.e., tips showing the Au(111) surface state were used for spectroscopy measurements on the molecules. The spectra were taken over several molecules and over many positions over the molecules, at a set distance above the molecules. Between measurements, the metallicity of the tip was confirmed by performing STS measurements of the bare metal surface to check for surface states. The resonances were ascribed to frontier orbitals after obtaining dI/dV maps of multiple molecules, allowing us to converge the STS peaks to the brightest contrast over the molecules.

## Supplementary Information

Below is the link to the electronic supplementary material.

Supplementary Material 1

## Data Availability

The datasets used and/or analyzed during the current study available from the corresponding author on reasonable request.
