# Supplementary material for: The effect of lateral π-extension on azulene-based molecules on surface studied by LT-STM
Source: Sci Rep. 2026 Apr 2;16:11226. doi: 10.1038/s41598-026-46150-4 (PMC13046993; doi:10.1038/s41598-026-46150-4)
Supplement: Supplementary file 1 — Supplementary Material 1 [file 41598_2026_46150_MOESM1_ESM.pdf]

Supporting Information

The effect of lateral π-extension on azulene-based molecules on surface studied by LT-STM

Suchetana Sarkar[1+], Natasha Khera[1], Kwan Ho Au-Yeung[1~], Renxiang Liu[1,2,3], Ji Ma[1,2,3], Xinliang Feng[1,2,3], Francesca Moresco[1]*

[1] Center for Advancing Electronics Dresden, TU Dresden, 01062 Dresden, Germany

[2] Chair of Molecular Functional Materials and Faculty of Chemistry & Food Chemistry, TU Dresden, 01062 Dresden, Germany

[3] Max Planck Institute of Microstructure Physics, Weinberg 2, Halle, Germany

Present address:

[†]Department of Chemistry, Philipps-Universität Marburg, 35032 Marburg, Germany

[~]Physikalisches Institut, Karlsruhe Institute of Technology, Karlsruhe, Germany

* Email: francesca.moresco@tu-dresden.de;

Table of Contents

Adsorption of CPAT on Au(111) 3

Adsorption of CPAT-Ph on Au(111) 4

Spectroscopy of CPAT on Au(111) 5

Scanning Tunneling spectroscopy of CPAT-Ph on Au(111) 7

Post-Annealing Results: CPAT on Au (111) 8

Post-Annealing Results: CPAT-Ph on Au (111) 9

Linescan Comparison of CPAT-Ph on Au(111) and Cu(110) 10

Post-Annealing Results: Isolated CPAT-Ph on Cu(110) 11

Adsorption of CPAT on Au(111)

The figure below shows an STM overview of the surface after deposition at RT, with the contrast suitably modified to highlight the herringbone reconstruction.

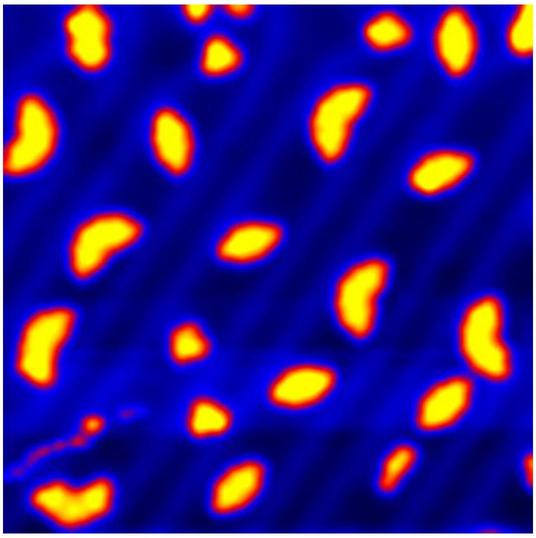

***Figure S1**. **CPAT** after deposition onto RT Au(111). Image parameters: V = 0.2 V, and I = 20 pA; size 20 × 20 nm²*

The figure below shows examples of self-assembled homochiral nano-architectures. The bright protrusions can be used as a guide to the eye to identify the difference in chirality.

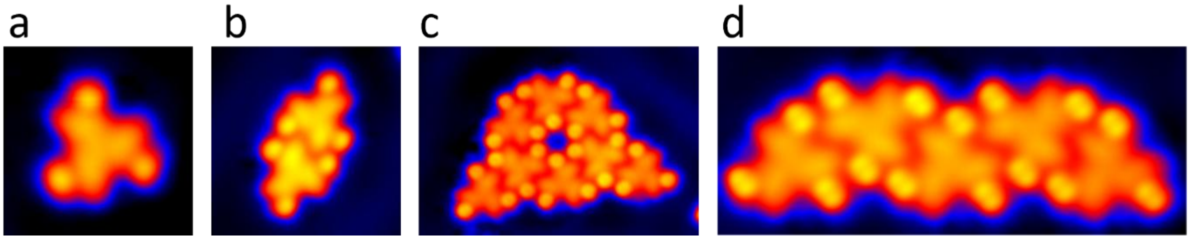

***Figure S2**. High Resolution STM images of self-assembled CPAT nanostructures on Au(111). (a) V = 0.2 V; I = 20 pA; size 2.9 × 2.9 nm². (b) V = 0.2 V; I = 20 pA; size 4.3 × 4.3 nm² (c) V = 0.2 V; I = 20 pA; size 8.5 × 5.7 nm²; (d) V = 0.2 V ; I = 20 pA; size 7.1 × 2.9 nm²*

Adsorption of CPAT-Ph on Au(111)

The figure below shows an STM overview of the surface after deposition at RT.

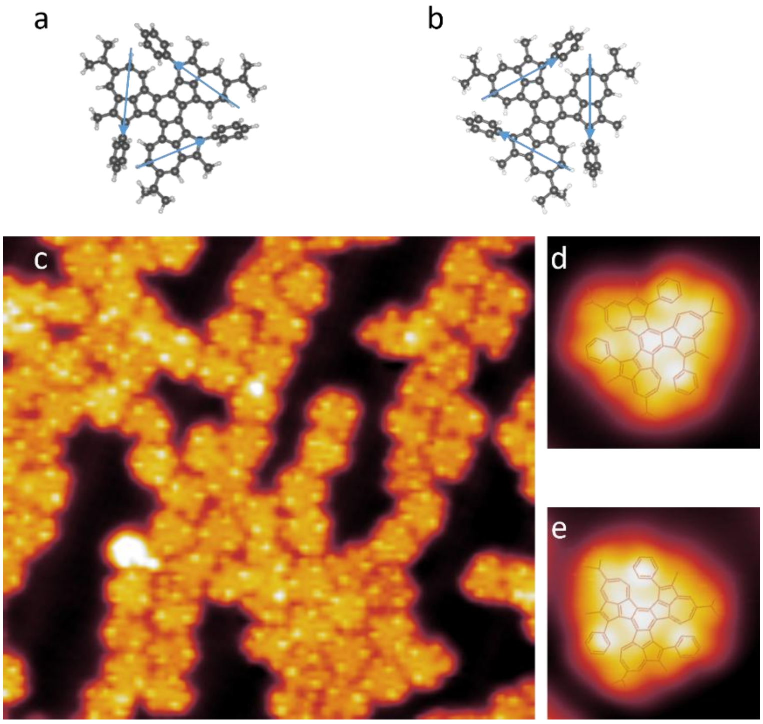

Figure S3: ***CPAT-Ph*** *after deposition onto RT Au(111). (a) and (b): The two different chiralities. The blue arrows show the orientation of the azulene units, which have been used to assign chiral notations. (c) Large area overview showing heterochiral assembly. Image parameters:* $V = 0.2$ *V and* $I = 20$ *pA; size 19 × 17* nm^2*. (d) and (e) Single molecules, each of a different chirality with the chemical structure superimposed. Image parameters:* $V = 0.2$ *V;* $I = 30$ *pA; size 2.5 x 2.5* nm^2*.*

Spectroscopy of CPAT on Au(111)

The figure below shows the spectra of **CPAT** molecule taken at the sides, where the resonance at -0.9V is better resolved.

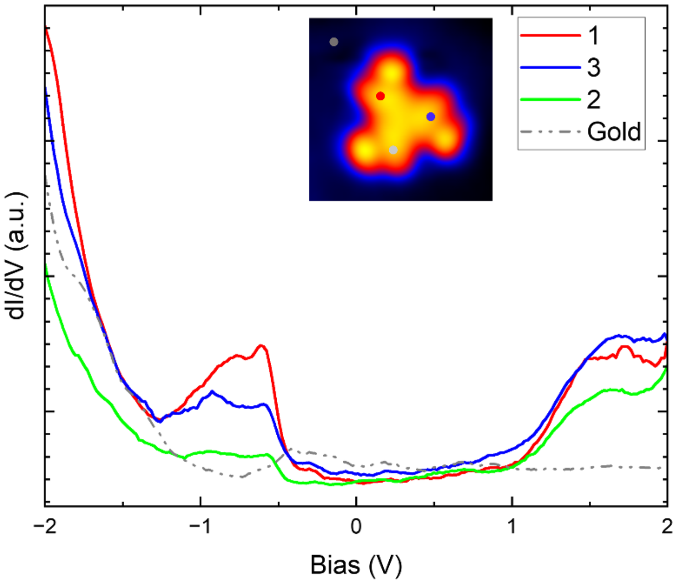

Figure S4. *Electronic properties of **CPAT** on Au(111): dI/dV spectra taken at the sides of a single molecule. Inset: V = 0.2 V; I = 20 pA; size 2 × 2 nm*^{2}.

The figure below shows spectra taken on a single molecule and molecule in a chain, to demonstrate the invariance of the electronic states going from single (main manuscript Figure 3), to dimers and chains.

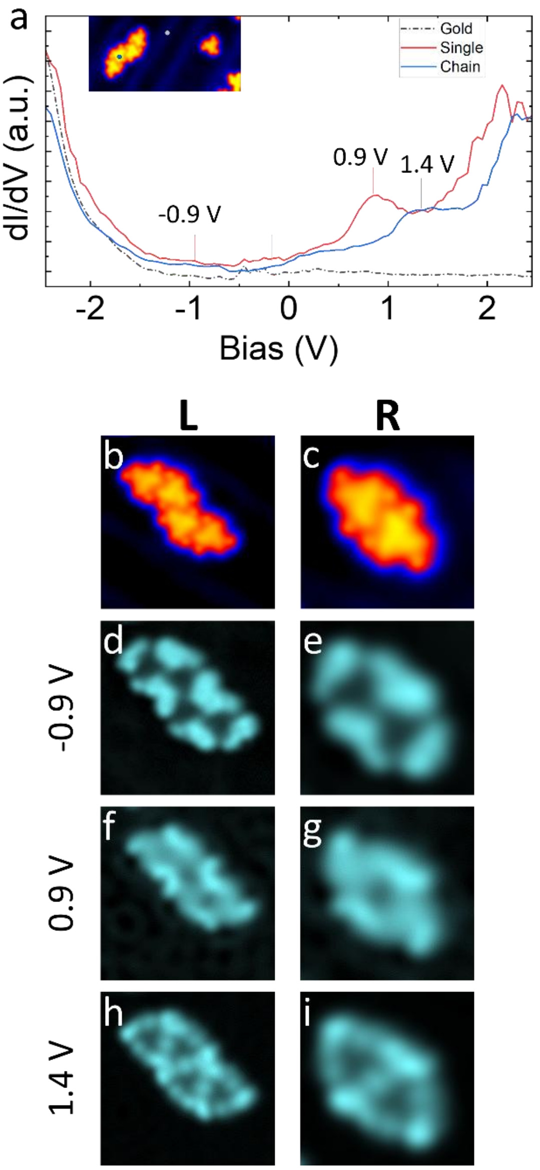

Figure S5. *Electronic properties of* ***CPAT*** *on Au(111). (a) dI/dV spectra on single molecule (red) and a molecule in chain (blue). Inset image: V = 0.2 V; I = 20 pA; size 12.5 x 5.7 nm*^{2}*. (b),(c) STM images of molecules adsorbed in chains and dimers, respectively. Image parameters: V = 0.2 V; I = 20 pA. (d)-(i) Corresponding dI/dV maps taken at different biases obtained from STS. Image parameters: I = 200 pA; size (b), (d), (f), (h) 5 x 5 nm*^{2}*, (c), (e), (g), (i) 3 x 3 nm*^{2}

Scanning Tunneling spectroscopy of CPAT-Ph on Au(111)

The figure below shows STS spectra taken along the sides of the **CPAT-Ph** molecule.

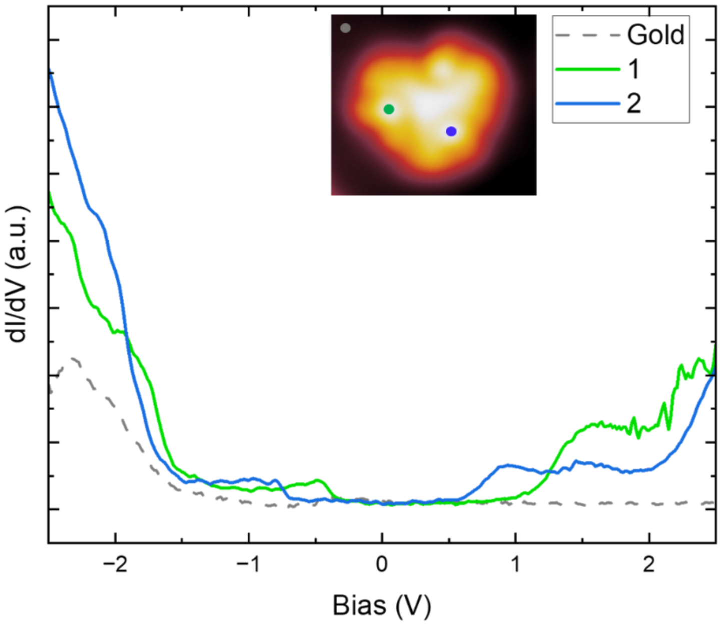

Figure S6. *Electronic properties of CPAT - Ph on Au(111). (a) dI/dV spectra taken at the sides of a single molecule. Inset: V = 0.5 V; I = 10 pA; size 3.5 x 3.5 nm²*.

Post-Annealing Results: CPAT on Au (111)

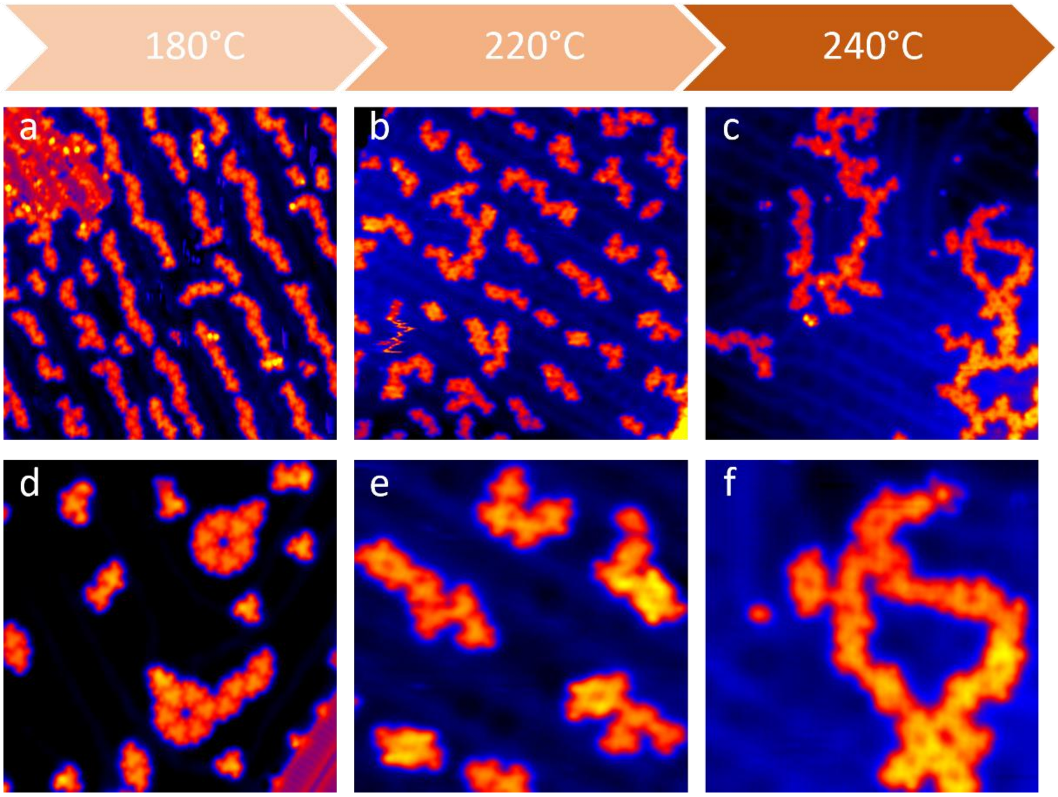

Figure S7: *Post-annealing* ***CPAT*** *on Au(111). (a)-(c) Large area STM overviews after annealing the* ***CPAT*** *molecules on Au(111) for 10 minutes at the specified surface temperatures. (d)-(f) Smaller area STM images showing desorption and disordered linkages. Image parameters: (a)-(c) V = 0.2 V; I = 20 pA; size 40 x 40 nm^2. (d)-(f): V = 0.2 V; I = 50 pA; size 15 x 15 nm^2.*

Post-Annealing Results: CPAT-Ph on Au (111)

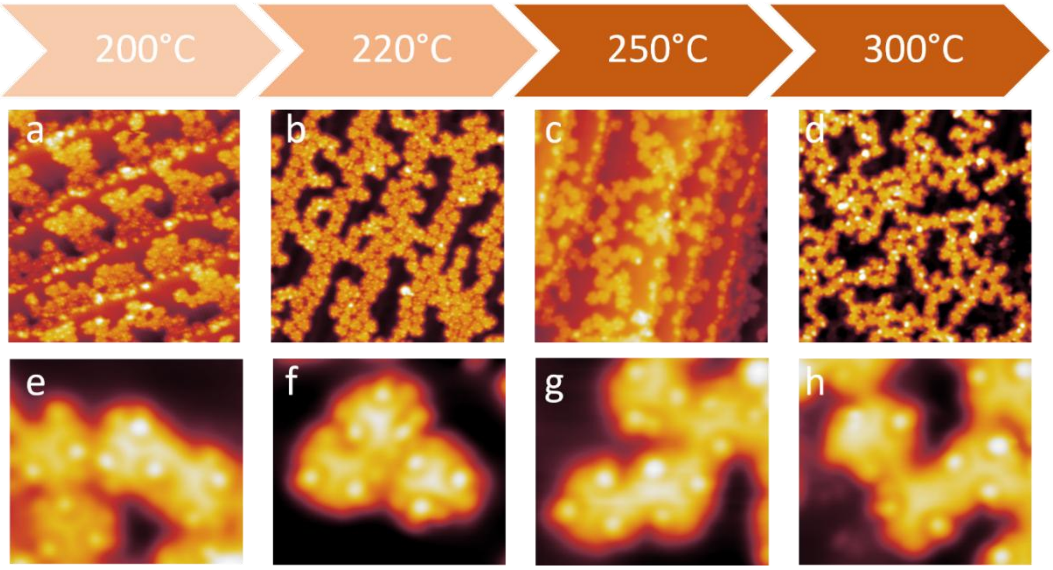

Figure S8: *Post-annealing* ***CPAT-Ph*** *on Au(111). (a)-(d) Large area STM overviews after annealing the* ***CPAT-Ph*** *molecules on Au(111) for 10 minutes at the specified surface temperatures. (e)-(h) Close up scans showing desorption and disordered linkages. Image parameters: (a) V = 0.2 V; I = 10 pA; (b) V = 0.2 V; I = 50 pA; (c) V = 0.2 V; I = 50 pA; (d) V= 0.5 V; I = 20 pA; (a) – (d): size 30 x 30 nm*^{2}*. (e) V = 0.2 V; I = 10 pA; (f) V = 0.5 V; I = 20 pA; (g) V = 0.2 V; I = 50 pA; (h) V = 0.5 V; I = 20 pA; (e) – (h): size 4.5 x 4 nm*^{2}*.*

Linescan Comparison of CPAT-Ph on Au(111) and Cu(110)

The figure below shows linescan profiles of **CPAT-Ph** molecule on Au(111) and on Cu(110) before and after annealing to 250°C.

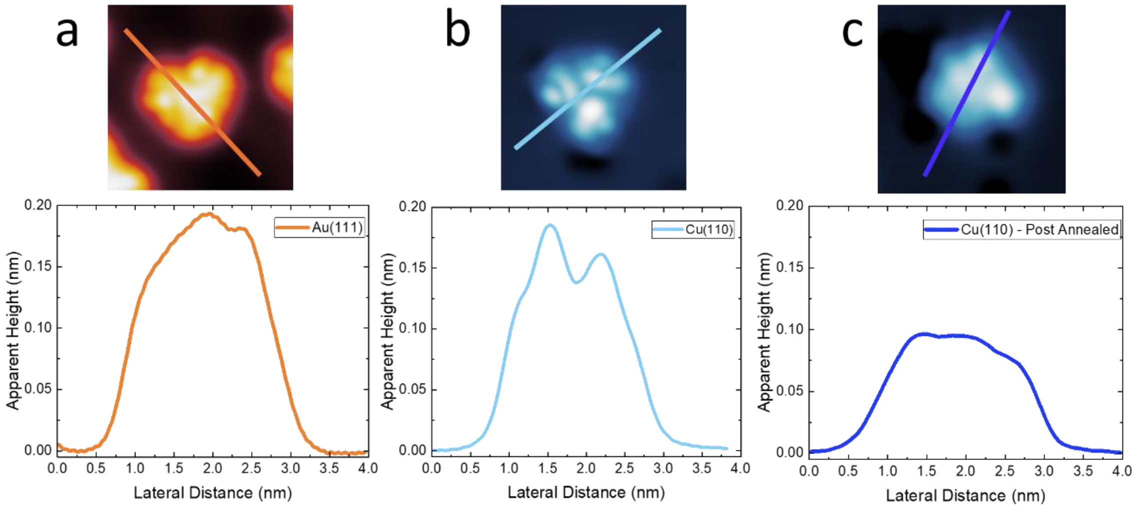

***Figure S9**: Line profile comparison of **CPAT-Ph** on Au(111) and Cu(110) (a) Line of profile on Au(111) (b) Line profile on Cu(110) (c) Line profile on Cu(110) post annealed to 250°C; Image parameters: (a) V = 0.2 V and I = 30 pA; size 4 × 4 nm^2, (b) V = 0.2 V and I = 10 pA; size 4 × 4 nm^2, (a) V = 0.2 V and I = 50 pA; size 4 × 4 nm^2*

Post-Annealing Results: Isolated CPAT-Ph on Cu(110)

The figure shows an isolated molecule that was found after post-annealing the sample to 250°C.

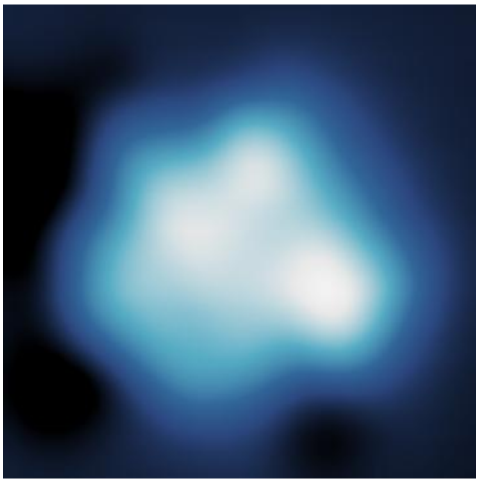

Figure S10. *Post-annealing* ***CPAT-Ph*** *molecules on Cu(110): Isolated planar molecule. Image parameters:* V = 0.2 V, I = 20 pA; size 2.5 × 2.5 nm^2.

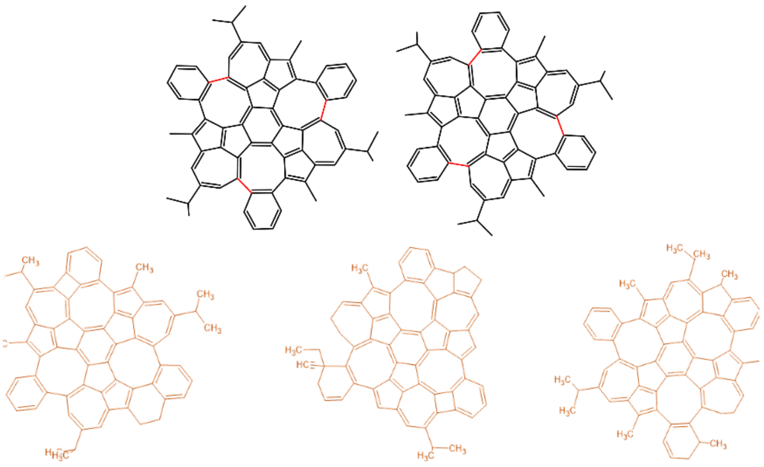

Figure S11*: Possible molecular structures post intramolecular ring closure reactions. The top row shows the ideal ring-closing reactions, while the bottom row shows probable ring closing reactions.*
